# A case of transvaginal NOTES partial gastrectomy using new techniques and devices

**DOI:** 10.1186/s40792-015-0093-6

**Published:** 2015-10-06

**Authors:** Shigeyoshi Higashi, Kiyokazu Nakajima, Yasuhiro Miyazaki, Tomonori Makino, Tsuyoshi Takahashi, Yukinori Kurokawa, Makoto Yamasaki, Shuji Takiguchi, Masaki Mori, Yuichiro Doki

**Affiliations:** Department of Gastroenterological Surgery, Osaka University Graduate School of Medicine, 2-2, Yamadaoka, Suita, Osaka 565-0871 Japan; Division of Next Generation Endoscopic Intervention (Project ENGINE), Global Center for Medical Engineering and Informatics, Osaka University, Suite 0912, Center of Medical Innovation and Translational Research, 2-2, Yamadaoka, Suita, Osaka 565-0871 Japan

**Keywords:** Natural orifice translumenal endoscopic surgery, NOTES, Transvaginal, Gastrectomy

## Abstract

The latest technique of transvaginal NOTES partial gastrectomy is described in detail. The procedure involves new “over-tube steering” technique and usage of two newly developed endoscopic accessories. The technique is feasible, safe, and practical, since all devices used in the case are off-the-shelf products.

## Background

Ten years have passed since the original concept of NOTES (Natural Orifice Translumenal Endoscopic Surgery) was proposed by Kalloo et al. [1]. The concept of intra-abdominal surgery using flexible endoscopy without causing major destruction of the abdominal wall was extremely innovative at that time; therefore, numerous preclinical feasibility/safety studies have been conducted by surgeons and gastroenterologists [2–6]. Although less postoperative pain and better cosmesis have been sporadically reported, its true less invasiveness has not been clearly described. The clinical application of NOTES thus has still remained in “exploratory” phase in limited advanced institutions, and various problems including medical, technical, ethical, and economical issues remain unsolved [3, 7, 8]. Is clinical NOTES already dead or alive? The authors believe that only a steady continuation of preclinical/clinical researches may address this question.

The authors formed a multi-disciplinary NOTES team with surgeons, gastroenterologists, and gynecologists and started conjoined preclinical research in 2006 [4, 6]. Our team then successfully performed the world’s first human transvaginal NOTES partial gastrectomy in a hybrid manner in 2008 [9]. To date, we have continuously improved our surgical techniques using commercially available instruments and newly developed devices [10]. In this communication, we describe our latest technique of transvaginal hybrid NOTES gastrectomy in a case with gastric gastrointestinal stromal tumor (GIST), using new techniques and newly developed endoscopic devices.

## Case presentation

**Fig. 1:**
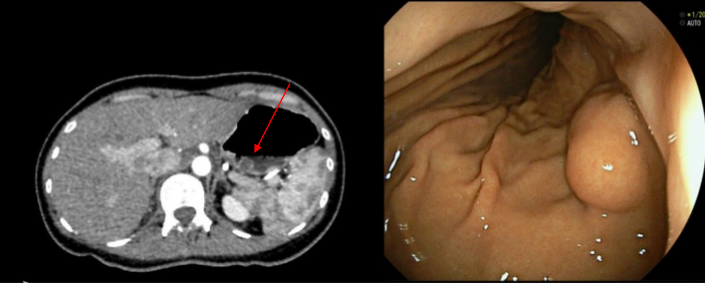
Abdominal computed tomography showing 2.5-cm submucosal tumor on the posterior wall of the gastric fornix with intra-gastric growth, and its endoscopic finding showing a distance of 4.0 cm between the lesion and the esophago-gastric junction

A 38-year-old female patient with an incidentally found gastric submucosal tumor was referred to our hospital for the consultation of the possibility of NOTES gastrectomy. The patient herself wished to undergo NOTES as a surgical alternative, due to possible less pain and better cosmesis. Her body weight was 41 kg and height was 155 cm. The patient was asymptomatic and otherwise healthy without any gastrointestinal disorders. The lesion, 2.5 cm in size, was demonstrated on the posterior wall of the gastric fornix by preoperative abdominal computed tomography. Endoscopy revealed a SMT with intra-gastric growth, with the distance of 2.0 cm below the esophago-gastric junction (Fig. 1). Biopsy was not attempted due to the size and location of the lesion. The patient experienced one childbirth with normal transvaginal delivery. After intensive discussion among the team, transvaginal NOTES partial gastrectomy was indicated as a surgical alternative. We informed the patient of its experimental nature, our previous clinical experience, the procedure’s short- and long-term outcomes, the possible risks/complications, and the details of the procedure itself. Surgery was conducted under the protocol approved by the University’s Institutional Review Board (IRB Folio No. 08063). Table 1 demonstrates our inclusion/exclusion criteria for transvaginal NOTES gastrectomy, which was approved by the University’s Institutional Review Board.

**Table 1:** Inclusion and exclusion criteria

Inclusion criteria
1. Tumor size <5 cm
2. Radicality by laparoscopic partial gastrectomy
3. Female and multipara
4. Age between 20 and 75 years
5. No history of gynecological diseases such as endometriosis and ovarian tumor
6. No history of abdominal surgery and lower gastrointestinal tract surgery
7. No history of diseases that cause pelvic adhesions, such as pelvic peritonitis
8. Provided written informed consent
Exclusion criteria
1. Patients who do not correspond to the inclusion criteria
2. Patients with severe comorbidity (liver disease, kidney disease, heart disease, respiratory disease, blood disease, metabolic disease such as diabetes, etc.)
3. Poor general condition
4. Performance status of 3 or more
5. Not fit to the protocol treatment by the physician’s decision

The routine part of our transvaginal NOTES technique has been described elsewhere [6, 7]. Briefly, after placing two laparoscopic ports at the umbilicus (5 mm) and the left mid-abdomen (3 mm), the posterior vaginal sac was exposed and punctured under laparoscopic guidance. This entry site was secured with an extra-long endoscopic over-tube, and a standard flexible gastrointestinal endoscope was advanced into the abdomen. The peri-gastric ligaments were dissected with energy devices passed through the endoscope to mobilize the involved portion of the stomach. The endoscope was then replaced with a linear stapling device. EGD was advanced trans-orally into the stomach to calibrate the EGJ and to obtain adequate surgical margin during stapling (so-called gastric calibration). Stapled partial gastrectomy was accomplished under laparoscopic monitoring. The specimen was isolated in a plastic bag and was delivered via the vagina. The vaginal entry site was closed with sutures under direct vision.

In this particular patient, we employed new techniques and newly developed accessory devices.

### New technique: over-tube steering using loop-retracting device

**Fig. 2:**
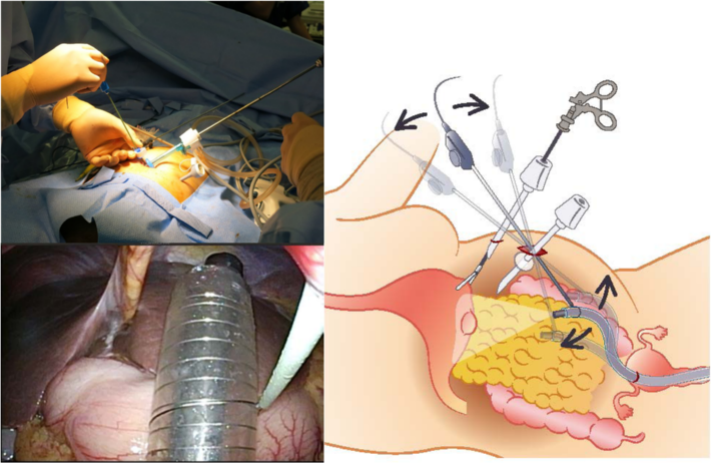
The over-tube being secured with a loop-retracting device for “over-tube steering technique”

The transvaginal over-tube was secured with a thin loop-retracting device (Mini Loop Retractor, Hakko, Nagano, Japan), which was introduced via the umbilical incision under retroflexed flexible endoscopic guidance. This technique enabled intentional “steering” of long and flexible over-tube in the abdominal cavity and greatly enhanced its stability while controlling the tip of the flexible endoscope (Fig. 2).

### New device 1: leak-proof adapter for endoscopic over-tube

**Fig. 3:**
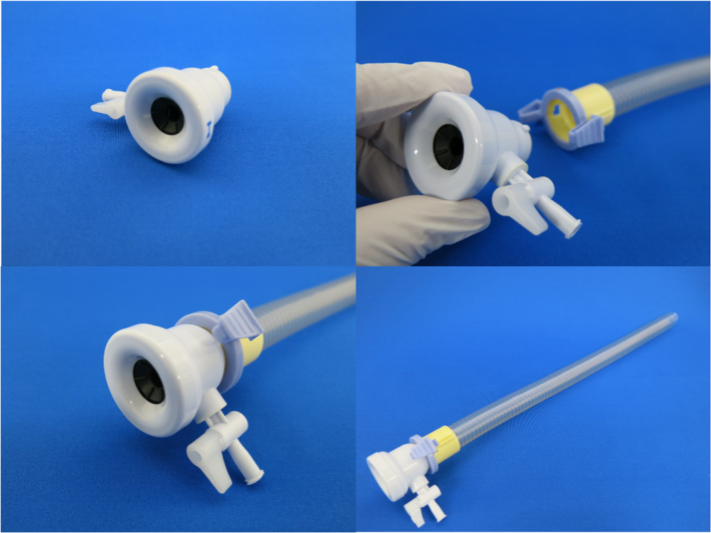
The leak-proof adapter (Leak Cutter) connected to a standard over-tube, to prevent major gas leakage from the distal orifice of the over-tube

Currently available over-tube has been designed to be used as a “guide” for flexible endoscope, and is not suitable to maintain pneumoperitoneum. Its distal orifice is not leak-proof; therefore, carbon dioxide, once fed through the trans-abdominal trocar, continuously leaks from the space between the over-tube and the endoscope. To solve this problem, we have developed a reinforced anti-leak adapter, and it was commercialized as an endoscopic accessory (Leak Cutter, Top Corporation, Tokyo, Japan) in 2012. With this simple device, pneumoperitoneum was successfully established and maintained all through the procedure without causing any major gas leakage (Fig. 3).

### New device 2: flexible irrigation/suction catheter

**Fig. 4:**
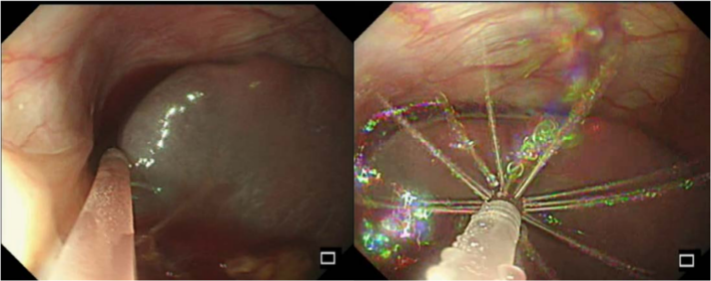
Use of flexible irrigation/suction catheter (Endoshower) for diffuse intra-abdominal irrigation using sterile saline solution, and atraumatic suction of the fluid in the left sub-diaphragmatic space under direct endoscopic vision

This newly developed device (Endoshower, Yamashina Seiki Co., Ltd., Siga, Japan) is a flexible catheter which passes the standard biopsy channel of current flexible endoscopes. Endoshower enables “laparoscopy-like” irrigation and suction under sterile condition. With this device, the abdomen was diffusely irrigated, and the fluid in the sub-diaphragmatic space was safely drained without causing any mis-suction and injuries on the splenic capsule (Fig. 4).

The procedure was successfully completed without any complications. No additional trocar was required, and no trans-abdominal energy device was used. The operative time was 260 min, and the blood loss was 20 ml. The patient showed uneventful and rapid postoperative recovery without any analgesic. She discharged from the hospital without any abdominal/gynecological complaints on the fifth postoperative day, which was determined by the IRB-approved clinical protocol. The postoperative pathology revealed leiomyoma of the stomach, 28 × 15 × 10 mm in size. Both umbilical and mid-abdominal scars are now well consolidated and virtually invisible.

### Discussion

One of the technical challenges in human NOTES is how to control and stabilize flexible endoscope in the abdominal cavity [2, 3, 7, 8]. Various commercially available/non-available over-tubes, i.e., platforms, have been attempted; however, virtually none of them have archived true clinical success [7, 8]. Our “over-tube steering technique” using loop-retracting device is simple, effective, and inexpensive; however, its true advantage in reducing operating time/blood loss was not shown in this single case report. This technique is applicable to any type of NOTES and practical since it is compatible with any kind of off-the-shelf endoscopic over-tubes. Further accumulation of clinical cases is definitely needed, to establish this technique as an attractive surgical option. The authors believe that future platform for pure NOTES should have at least “anchoring” mechanism that anchors the platform onto the abdominal lining.

How to maintain pneumoperitoneum is another fundamental but difficult problem in NOTES [5, 11]. Since commercially available endoscopic over-tubes are not designed to maintain a steady-pressure environment, they are not as leak-proof as laparoscopic trocars. Our newly developed “Leak Cutter,” now available in Japanese market as a reinforced anti-leak adapter for routine endoscopy, works perfect to prevent gas leakage during the case. The device has a Luer lock-type stopcock for emergent evacuation in case of over-insufflation. This stopcock can also be used as an insufflation inlet in future pure NOTES, where trans-abdominal insufflation via laparoscopic trocar will no longer be available.

The intra-abdominal irrigation via current flexible endoscope is feasible; however, it is technically demanding due to its one-direction spray from the tip of the endoscope. The use of sterile saline solution via built-in air/water channel requires specific and cumbersome preparation and setting. The suction from the tip of the endoscope is further challenging, since conventional technique requires “diving” of the endoscope beneath the fluid. While diving the endoscope, the visualization is temporally lost. This is acceptable in the gut lumen where no other organs exist; however, this is not acceptable in the abdominal cavity where blind mis-suction may lead to serious organ damage. Our new product, Endoshower, is one of the solutions. This device, 2.6 mm in outer diameter, has 24 side holes of 0.4 mm in size at its tip. With this device, sterile irrigation and safe suction under endoscopic visual control were feasible in the present case.

## Conclusions

Transvaginal hybrid NOTES partial gastrectomy has been refined with several new ideas and devices. Clinical NOTES is still in the early developmental stage. Continuous efforts of medical device development and further accumulation of clinical experiences are necessary to make NOTES a “reality” rather than a “dream.”

## Consent

The patient has provided permission to publish these features of her case, and the identity of the patient has been protected.
